# An Upgrade Pinning Block: A Mechanical Practical Aid for Fast Labelling of the Insect Specimens

**DOI:** 10.3897/BDJ.5.e20648

**Published:** 2017-10-09

**Authors:** Mohammad Hossein Ghafouri Moghaddam, Mostafa Ghafouri Moghaddam, Ehsan Rakhshani, Azizollah Mokhtari

**Affiliations:** 1 Department of Electrical, High Education Institute of Hatef, Branch of Zahedan, Zahedan, P.O. Box: 98155– 1579, I. R., Iran; 2 Department of Plant Protection, College of Agriculture, University of Zabol, Zabol, P.O. Box: 98615–538, I. R., Iran

**Keywords:** Mechanical pinning block (MPB), Entomology, Collections, Museums.

## Abstract

A new mechanical innovation is described to deal with standard labelling of dried specimens on triangular cards and/or pinned specimens in personal and public collections. It works quickly, precisely, and easily and is very useful for maintaining label uniformity in collections. The tools accurately sets the position of labels in the shortest possible time. This tools has advantages including rapid processing, cost effectiveness, light weight, and high accuracy, compared to conventional methods. It is fully customisable, compact, and does not require specialist equipment to assemble. Conventional methods generally require locating holes on the pinning block surface when labelling with a resulting risk to damage of the specimens. Insects of different orders can be labelled by this simple and effective tool.

## Introduction

Natural history museums (and even some personal collections) stand out as some of the most invaluable repositories of biological information. They are an important resource for preserve voucher specimens underpinning studies in taxonomy, ecology and related fields (Winker 2004, Turney et al. 2015). The principal objective of a natural history museum (NHM) is to provide the researchers, scientists, students with accurately identified recent and historical specimens for research. Museums have many diverse science programs including taxonomic studies on animal specimens, digitisation, and preparation of specimens, education, exhibitions, etc. In order for NHMs to be useful it is necessary to correctly arrange the specimens and provide easy access to their associated metadata, most often recorded on the specimen labels. The position, and end ease of reading of specimen labels is therefore important to the usefulness of biological collections. These specimens are preserved in different ways (ethanol, microscope slides, point card and plastic envelopes). Entomological specimens are primarily stored mainly dry-mounted or dry-pinned. Millions of pinned insect specimens are housed in natural history collections on a global scale. For example, the pinned insect collection of the Canadian National Collection of Insects (CNC) contains approximately 16 million specimens systematically arranged in 1400 steel cabinets (see Canacoll 2016). Gibb (2014) stated that: “***If reference materials are unorganized or difficult to access, they will not be used regularly and their value depreciates measurably***”; therefore, the value of standard specimens for insect collection provides optimum protection and ease of access for the valuable specimens and make the observation of individual specimens easy without actually removing them each time.

The final process for insert information mounted insects is to prepare and mount the labels in a practical arrangement for the collection. The primary purpose of a pinning block is to enhance the ability of people (students, researchers, curators) for make the pinning of insects, quicker, more precise and easier. Pins with specimens are held vertically during the labelling procedure. Therefore, if some researchers only wish to use traditional pinning blocks, it is suggested to use an alternative tools instead, which would be much simpler for curators and technician museums (e.g. see BioQuip co.or  www.bioinsectequipment.com); or even a flexible model of different colours made from plastic with a matte finish and slight grainy feel (see  http://www.shapeways.com/product/MXPXFP3AS/insect-pinning-block).

The role of the pinning block is to set specimens (card-mounted and/or pinned) and labels at proper heights on the pin with negligible damage and bending. It is crafted precisely to guarantee uniform pinned specimens with labels mounted in appropriate heights that can help to standardise the collection. It can be used to pin insects of various sizes simultaneously. The block therefore negates damage caused by pushing a mounted specimen into a flexible surface such as Styrofoam. A pinning block has a series of holes of different depths drilled in it to mount specimens and labels at standard heights. For the most part, the 3-step pinning block is made of walnut wood, with rounded ends along with plastic veneer on top to prevent damage from pin points and/or with a steel, aluminum, plastic, etc. and height for points or double mounts is set at 23 mm (1st the hole). The locality (2nd hole) and determination (3rd hole) label heights are set at 12 mm and 7 mm, respectively. Insect specimens are usually set for 13 mm height from top of the pin. Three holes are drilled through the side of the block with the above-mentioned sizes Borror and White 1998 to allow clearing of blockages to the holes.

Pinning blocks are released in many different shapes and sizes (Fig. 1). They can be made of simple wooden design or involve expensive steels with comparatively high cost. They are often, stair-step with 3 holes is commonly used in the laboratories and personal collections. A professional pinning block and using 5 holes with a steel cover, is used at many of the public museums of the world. A method that was developed by Borror and White (1998) and Walker and Crosby (1988) concerning stair-step and steel pinning block, and it is suitable to provide a more detailed description. Five-step and conventional pinning block, is made of steel, metal and wood with super hard coating, which permits continued heavy use without changing the label spacing. The 7, 12, 17, and 22 mm dimension holes can be used a labels, and the 27 mm hole for points (Walker and Crosby 1988). The price of 'Insect pinning block' varies in different stores from USD 3.0 to USD 39 (BioQuip co., Amazon, eBay, Bioinsectequipment, Carolina, etc.). Various types of insect pinning blocks are shown in Fig. 1; such as wooden, steel, aluminum, 3D, extravagant model, etc.

The new tools to mount specimens and labels to uniform height portrayed here were initially conceived by the last two authors and complex practiced with success on parasitoid wasps specimens for numerous years. Subsequent updates and improvements was implemented by the first author. The new cost effective, light weight, and high quality commercial product is presented here, and compared to previous designs of pinning blocks by the first author.

## Material and methods

To build this tools, measurements and primary design were made using Autodesk^®^ AutoCAD^®^ 2016 version M.49.0.0 software (Fig. 2). The initial and final animations for the original design of the tools were made using 3D Max^®^version 7.0 software. The materials used to build the tools are of poly methyl methacrylate (PMMA), also known as acrylic or acrylic glass (under the trade mark of Plexiglas^®^). PMMA technology has the proper attributes based on the standard of the current study. One sheet of Plexiglas^®^ with dimension of 115x115 mm was cut into several pieces using a CNC machine. The assembly procedures are shown in Fig. 3. Photographs were taken using a SONY^®^ Cyber-shot DSC-WX7 digital camera, then were slightly edited and assembled in Adobe Photoshop^®^ CS6.

**Figure 4.**
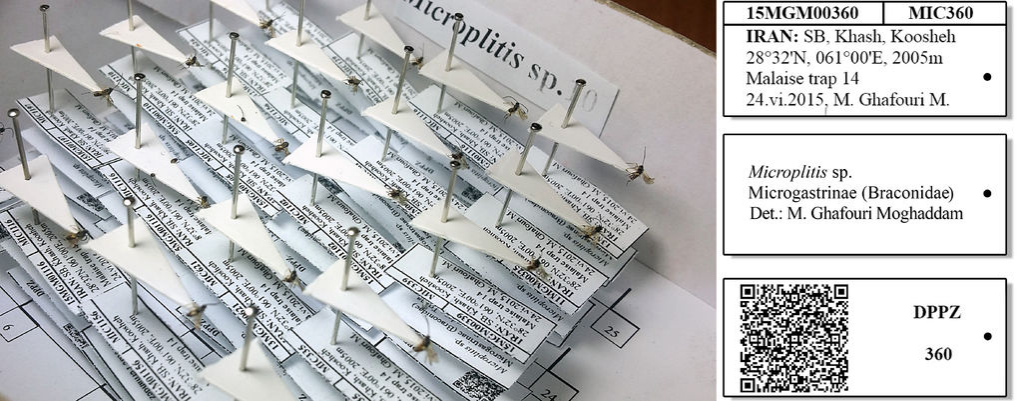
Specimens *Microplitis* sp. (Hymenoptera, Braconidae, Microgastrinae) arranged and labelled by upgraded pinning block in DPPZ.

For the quick access to details in the final version of the paper (*.pdf version) files *.DWG using Delcam Exchange^TM^ 2015 version 8.4.1004 software was used to convert DWG files (a file AutoCAD^®^) into U3D, that were then imported into Adobe Acrobat^®^ Pro DC XI ver. 11.0.09 (see Suppl. material 3). The animation controls (in all 3 dimensions: X, Y, and Z) were mapped to the mouse cursor. More details and complete explanation on the method of creation of this pinning block are provided as supplementary files (see Suppl. materials 1, 2, 3). A species, *Microplitis* sp. (Hymenoptera, Braconidae, Microgastrinae), was selected to test the applicability of the new tools and result that shown in Fig. 4. This specimen is deposited in the **D**epartment of **P**lant **P**rotection, University of **Z**abol, Zabol, Iran (**DPPZ**). The comparison of the important features of models pinning block in the worldwide are summarized in Table 1 and analyzed and selected the best rating.

The design of the Mechanical Pinning Block (MPB) model and step by step manufacturing are shown in more details in Figs 2, 3and Suppl. materials 1, 2, 3. Before using this tool, labels of each specimen of insects should be prepared and cut (Fig. 4). Information labels for specimens include location, geographical, Global Positioning System (GPS), barcode, etc. (see more details in Krogmann and Holstein 2010, Dellinger et al. 2016). Also, specimens are mounted on triangular cards and pinned using AXA (alcohol-xylene-amyl acetate) methods (van Achterberg 2009). Authors used 3.8 mm size pins, which can be changed into various sizes. After preparation of specimens and labels; first the labels are put in their place on MPB, preferably done with forceps not to be bend labels, then the specimens are mounted from above and pushed into the hole. Finally, using the rotation lever (embedded on the left side of the tools) specimens will be out with their label.

## Results

All the different tested pinning blocks can be used to produce regular labelled pinned specimens. Our MPB tool however achieves this in less time (see Suppl. material 4) due in part to a single insertion of the pin into the block. This tools is capable of accommodating insect specimens 1 to 3 mm in length, but in other models the amount is increased to facilitate the labelling of insect of various sizes. Further advantages of the MPB are its low cost, light weight, universal applicability, high quality and portability.

## Discussion

Conventional pinning blocks are generally time-consuming and risky, which can be detrimental to specimens. The updated pinning block is a time-expedient technique which reduces risk to specimens. Currently the MPB can be used only for insects with small size, making it impractical for some orders such as Lepidoptera. In general, these tools cannot be used for insects with large size. A proper label increases the scientific value of a specimen because it provides the name of the insect and other information.

MPB is also ideal and perfect for quick arrangement to increase the visibility of specimens and labels by curators and student taxonomists, thus increasing the aesthetics and usability of the collection Gibb and Oseto 2006. The MPB can be used to ensure that card points and labels are at uniform and appropriate heights through the collection; ensuring that there is sufficient room above the specimen to handle the pin without contact with the specimen.

Approximately 90% of the time required for digitisation is spent on capturing metadata and the labelling specimens; while the latter involves physical handling of the specimens and should be performed by experienced staff (Blagoderov et al. 2012). Therefore, the organisation and arrangement of the specimens and labels are a real goal for NHMs.

Based on the exhibited results (see final analysis in the Table 1), it can be presented that the new pinning block as it is currently used has improved or equal characteristics to standard pinning blocks. According to Table 1 pinning blocks performance rated from excellent to poor is: mechanical PB ≥ Rectangular steel ˃ rectangular wooden ≥ traditional wooden stair-step ˃ hardwood 5-steps ˃ extravagant model ˃ flexible stair-step, respectively.

A further advantage of the MPB is that each component part is easy to replace, either for repair or when updated materials/designs are available. Authors are pleased to receive ideas and feedback of readers for the next generation pinning blocks. It is an honor to advance, accelerate, and provide excellent results in specialized tasks and projects, especially project digitisation specimens in the museum, essential supplies are provided to entomologists.

## Supplementary Material

Supplementary material 1 Initial animation tools Data type: MOV file (*.MOV).
Brief description: Before initial design by the AutoCAD program we created an initial animation using the 3D Max program for our the better understand and draw in order to operate the project, in other words visualizing initial plan.
File: oo_152158.mov Mohammad Hossein Ghafouri Moghaddam, Mostafa Ghafouri Moghaddam, Ehsan Rakhshani and Azizollah Mokhtari

Supplementary material 2 Initial design and measurements Data type: ZIP file (*ZIP).
Brief description: This supplementary files is containing two files. We for to make this tools first designed an initial plan in AutoCAD program. Then each of the designed segment was put together on one page. Finally, the final file using the computer personal transferred to the CNC machine that to perform the cutting operation on the Plexiglas.
File: oo_152164.zip Mohammad Hossein Ghafouri Moghaddam, Mostafa Ghafouri Moghaddam, Ehsan Rakhshani

Supplementary material 3 Interactive model Data type: Adobe PDF file (*PDF).
Brief description: To view details of the tools from different angles and to handle screen transitions and image swapping that an animation file was embedded in the Adobe Acrobat program.
File: oo_152179.pdf Mohammad Hossein Ghafouri Moghaddam, Mostafa Ghafouri Moghaddam, Ehsan Rakhshani

Supplementary material 4 Video demonstration Data type: MOV file (*.MOV).
Brief description: This video shows how to work with the tools. Also, how to labeling, pinning and detail the tools is well illustrated.
File: oo_152180.mov Mohammad Hossein Ghafouri Moghaddam, Mostafa Ghafouri Moghaddam, Ehsan Rakhshani

## Figures and Tables

**Figure 1a.**
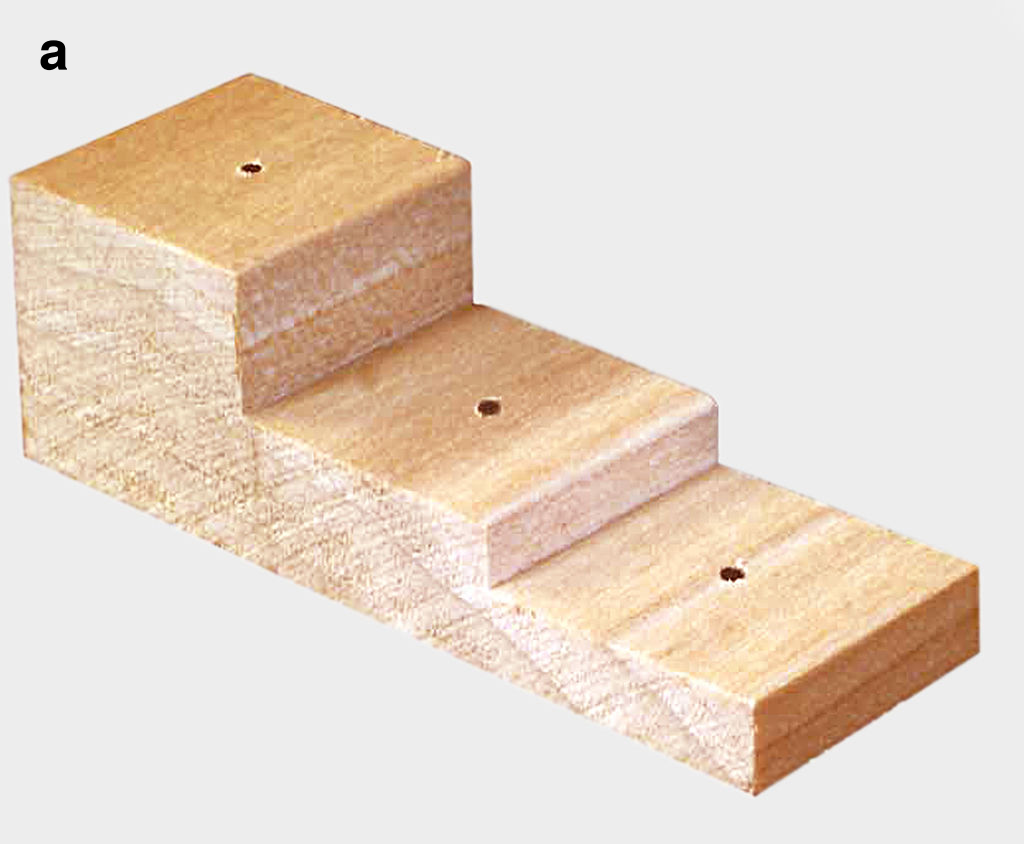
Traditional stair-step (BioQuip co.)

**Figure 1b.**
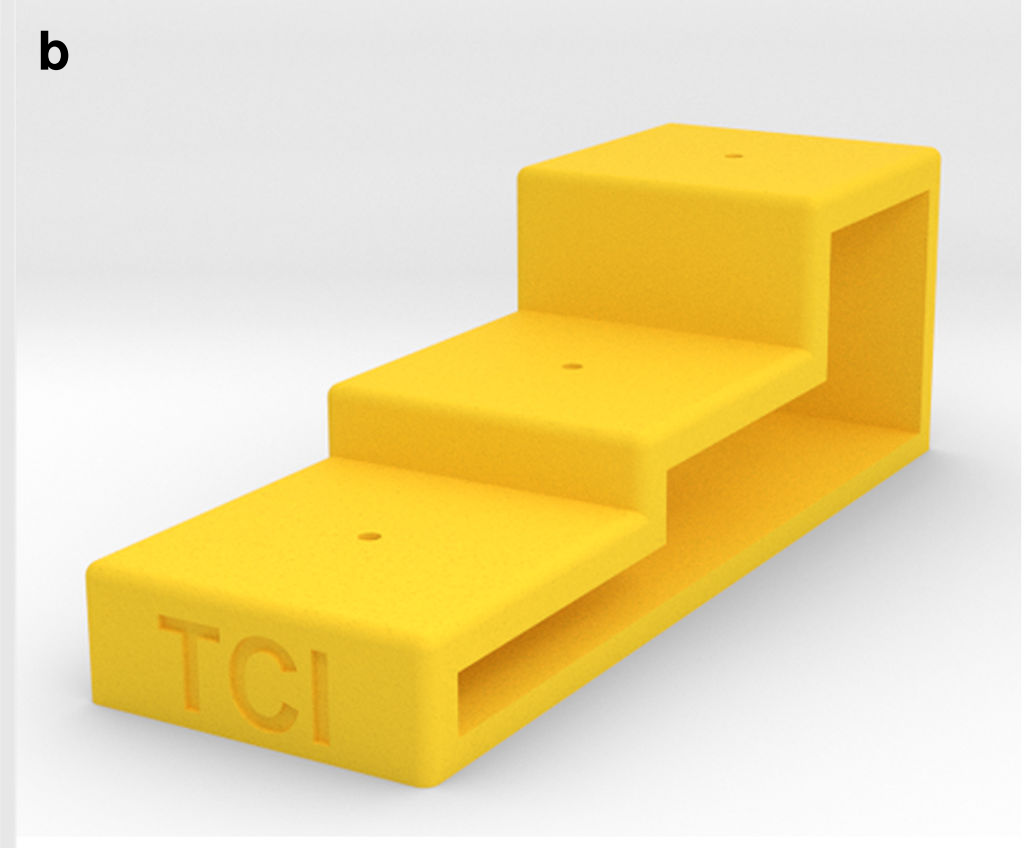
Flexible stair-step (http://www.thingiverse.com)

**Figure 1c.**
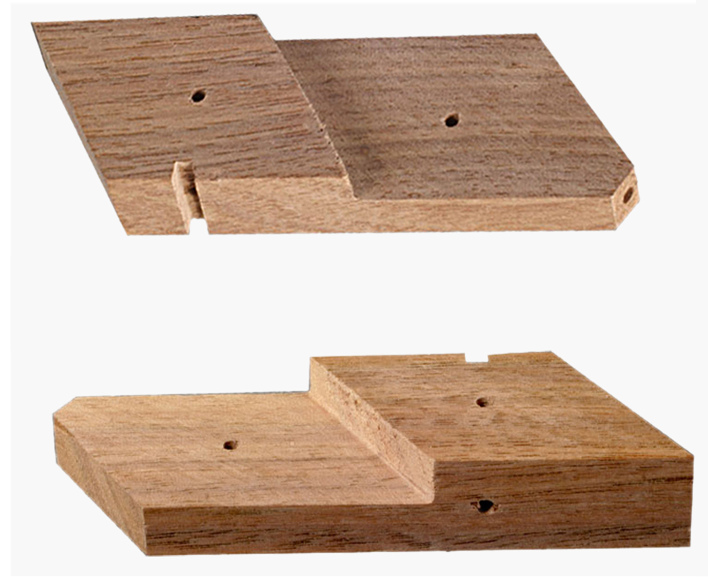
Hardwood with five stair-step

**Figure 1d.**
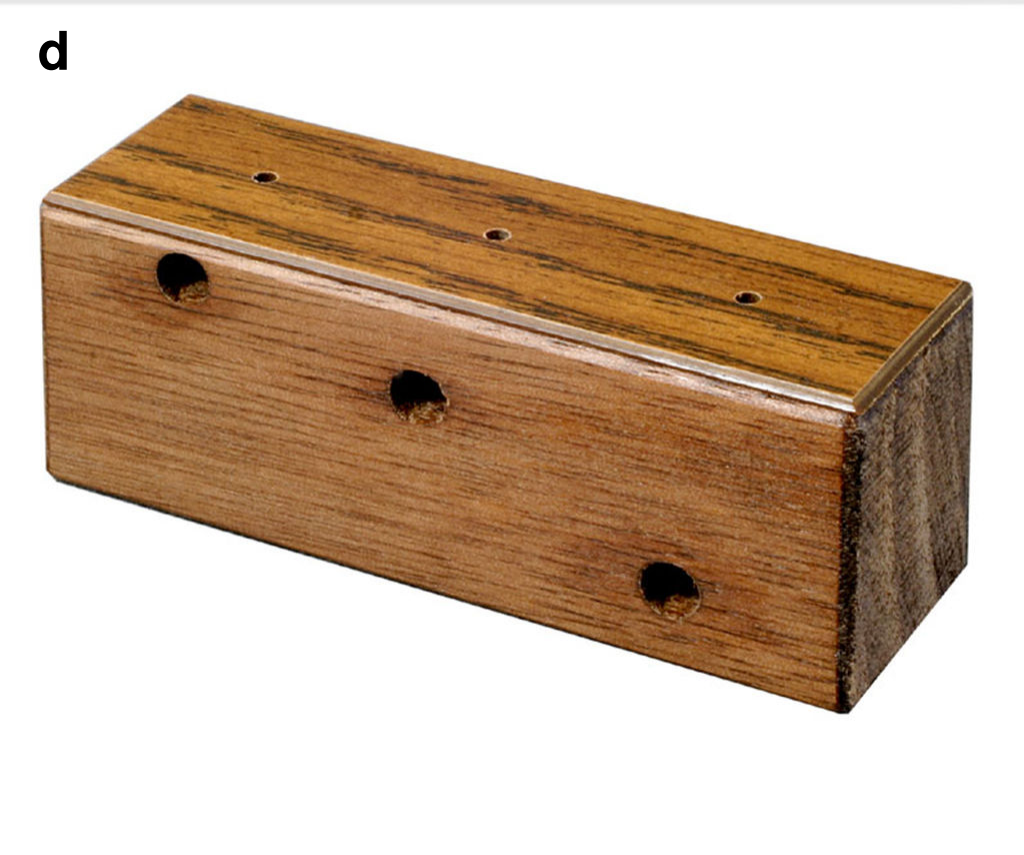
Rectangular wooden (BioQuip co.)

**Figure 1e.**
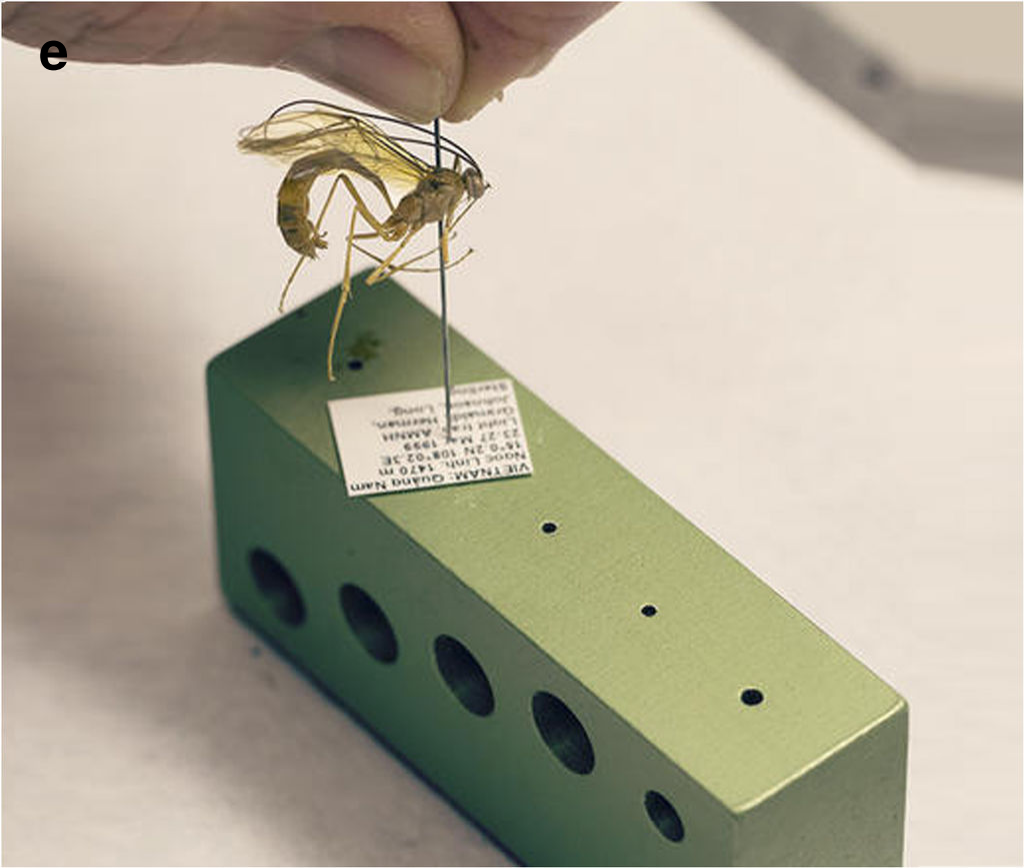
Rectangular steel (www.amnh.org)

**Figure 1f.**
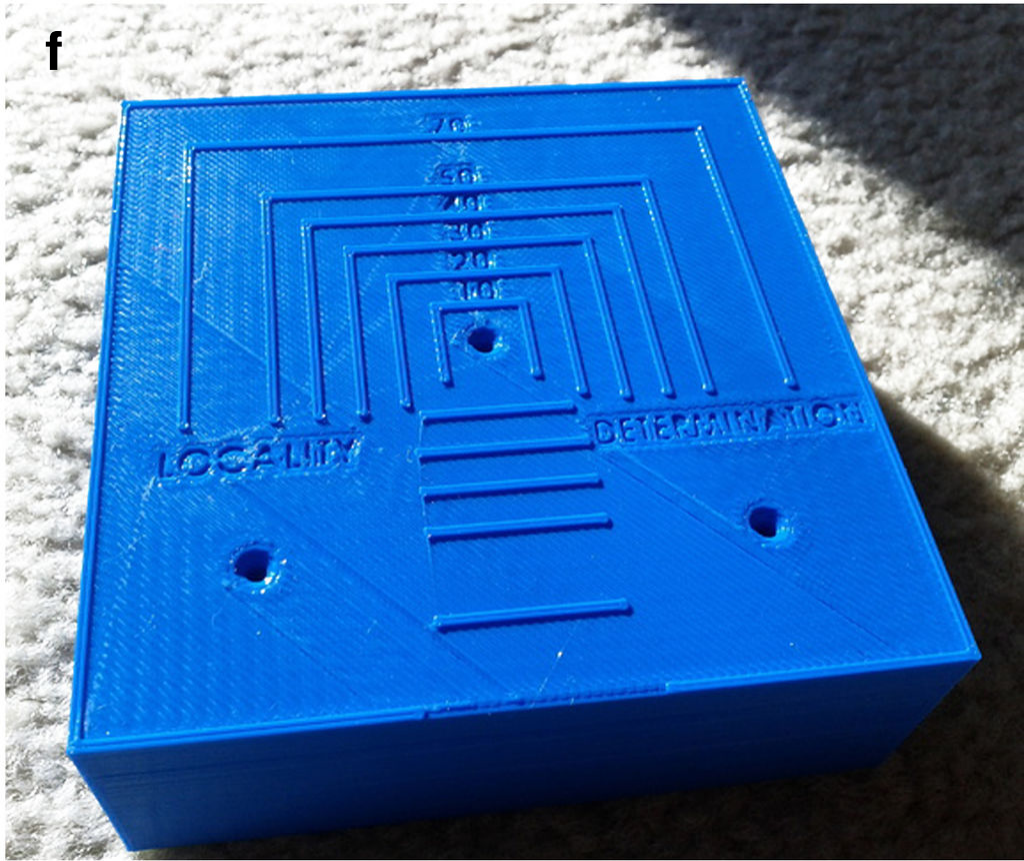
Extravagant model (http://www.thingiverse.com)

**Figure 2a.**
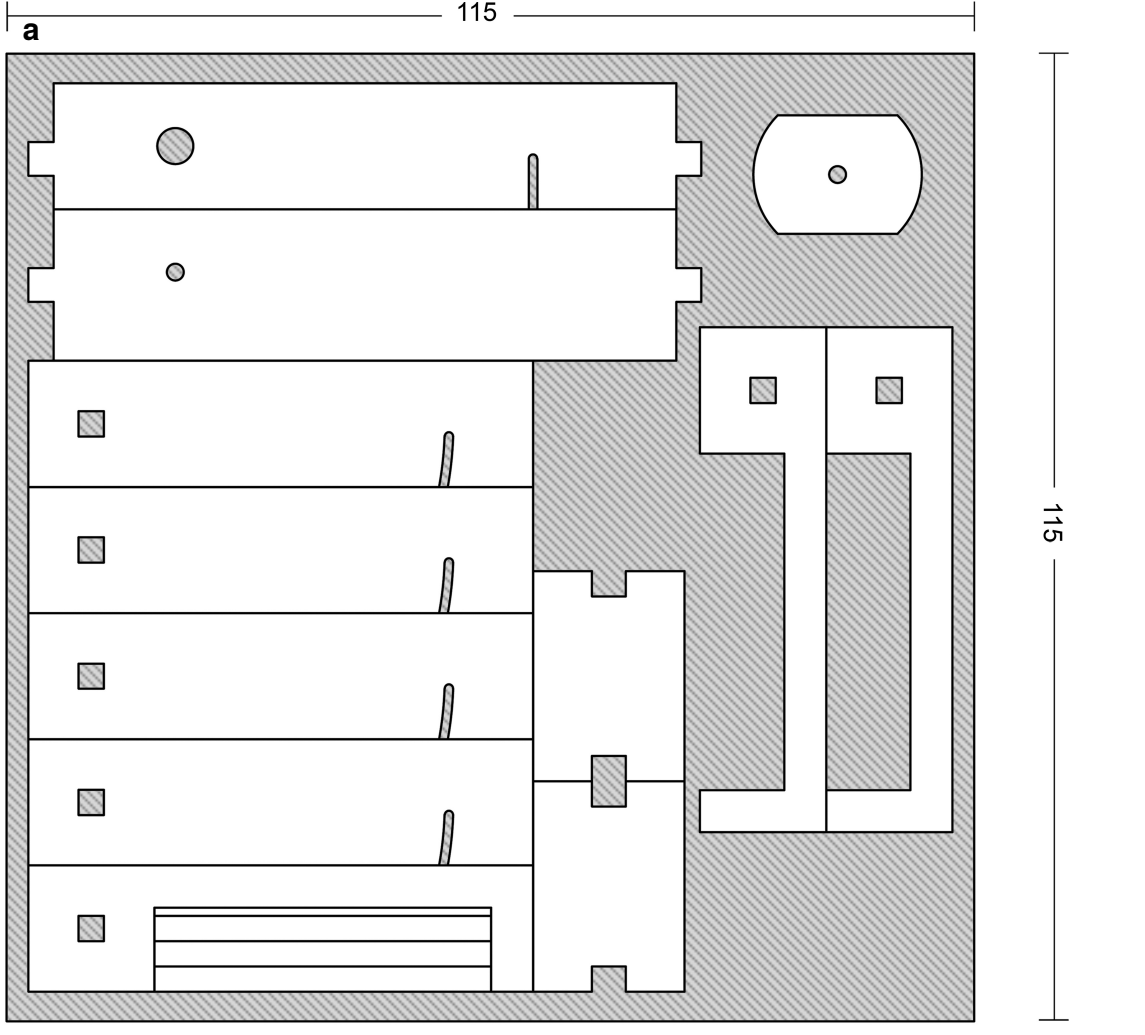
Primary design of updated pinning block (provided by Autodesk^®^ AutoCAD^®^ 2016)

**Figure 2b.**
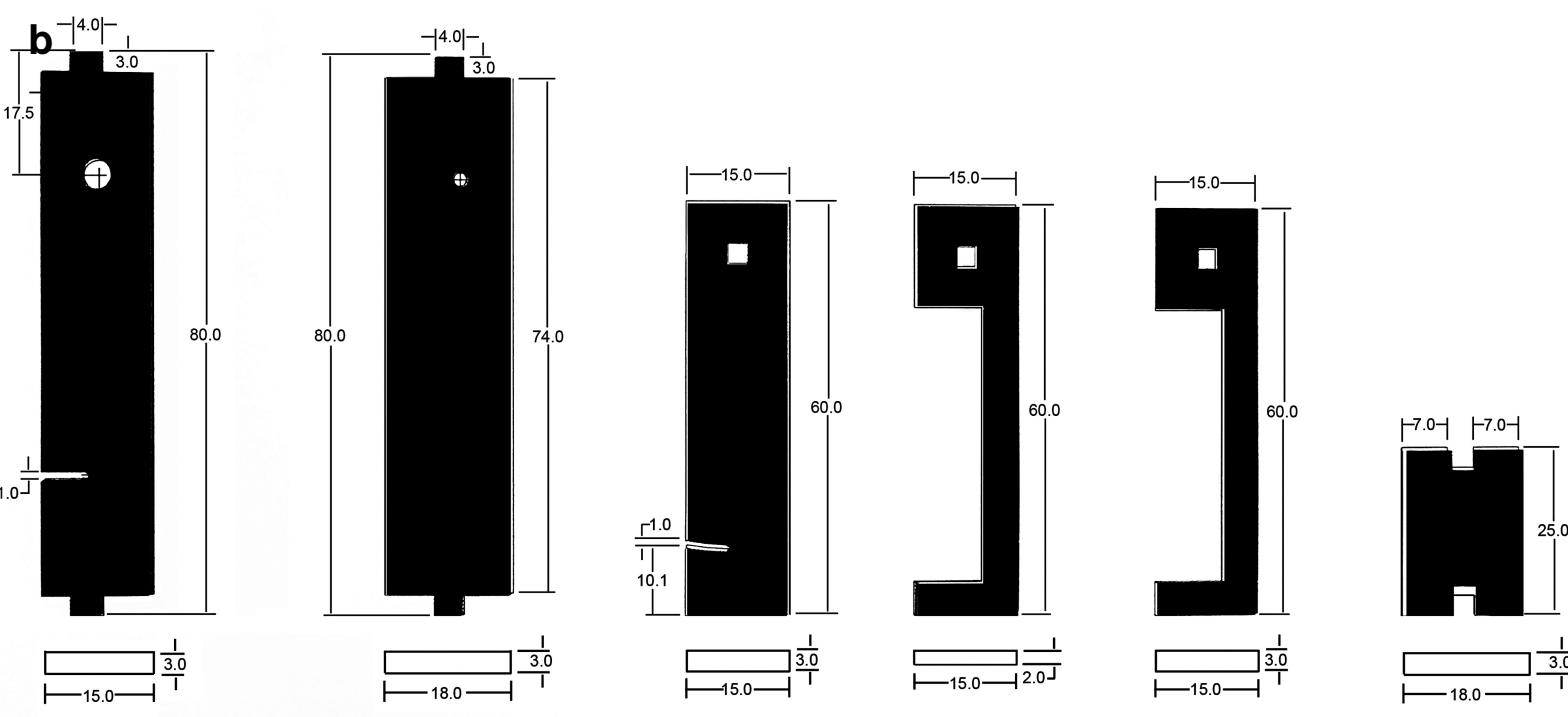
Details of measurements various components (in millimeters)

**Figure 3a.**
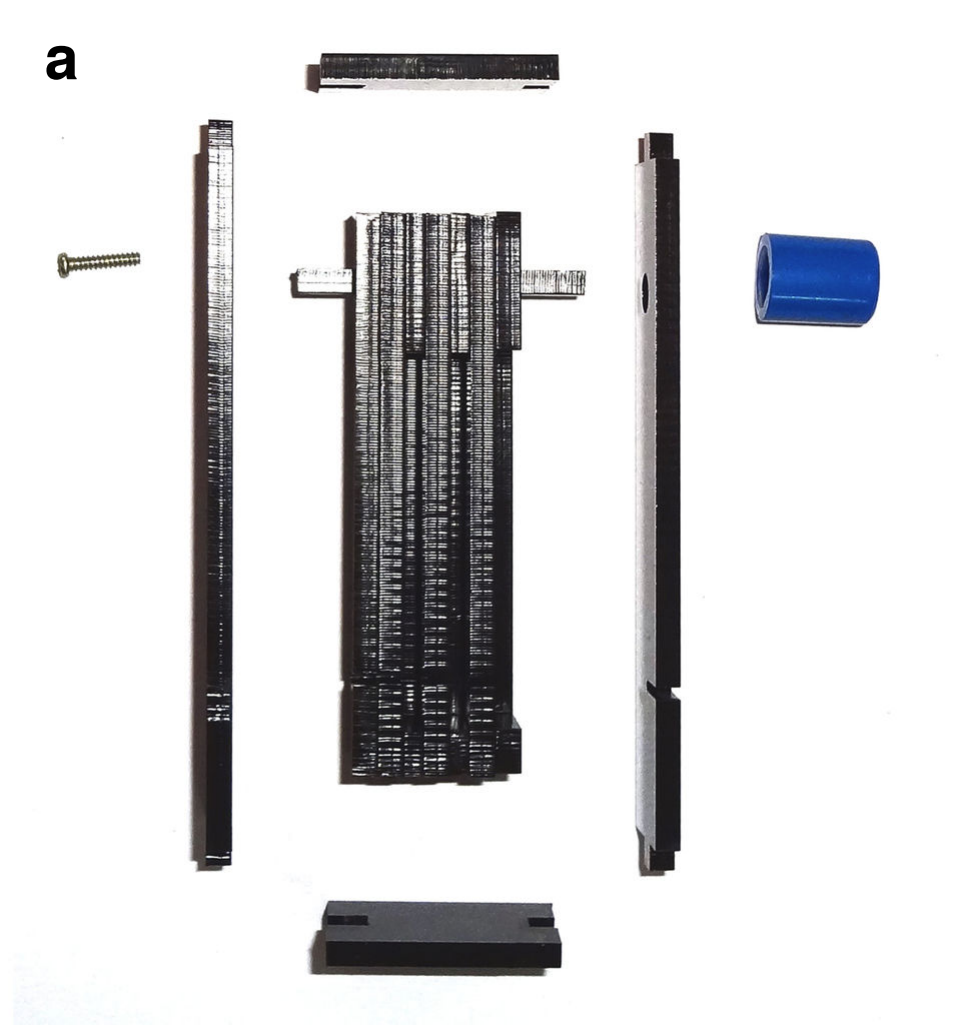
How this segment should be attached following connecting and fixing (lateral view)

**Figure 3b.**
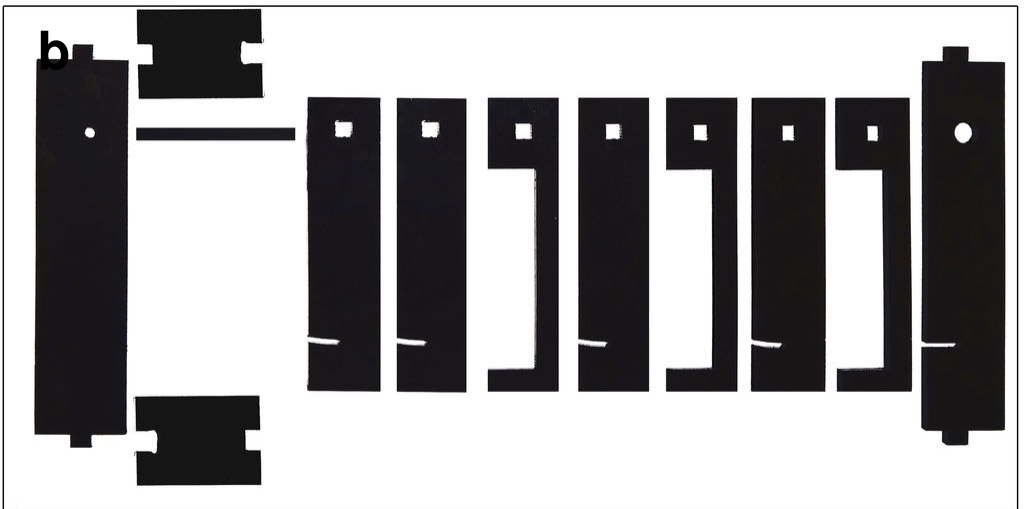
Detail of the Plexiglas^®^ segment (top view)

**Figure 3c.**
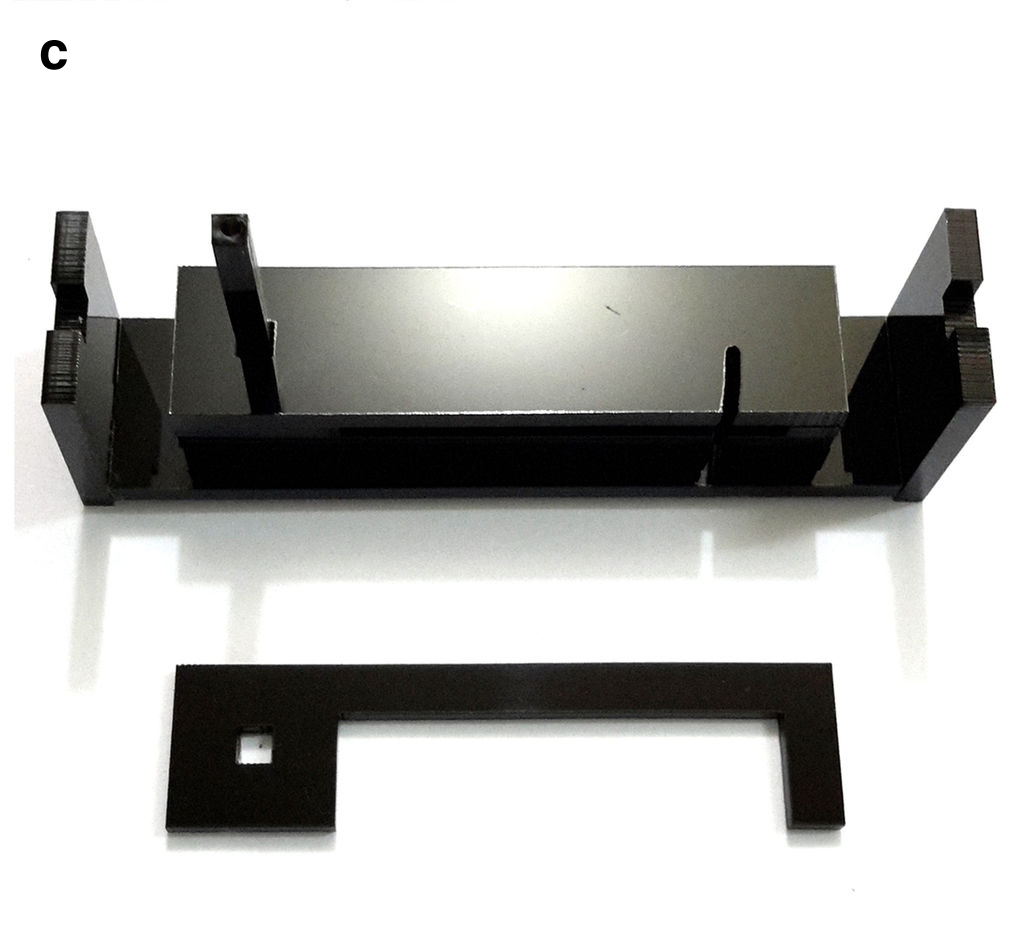
Assemble Plexiglas^®^ segment together

**Figure 3d.**
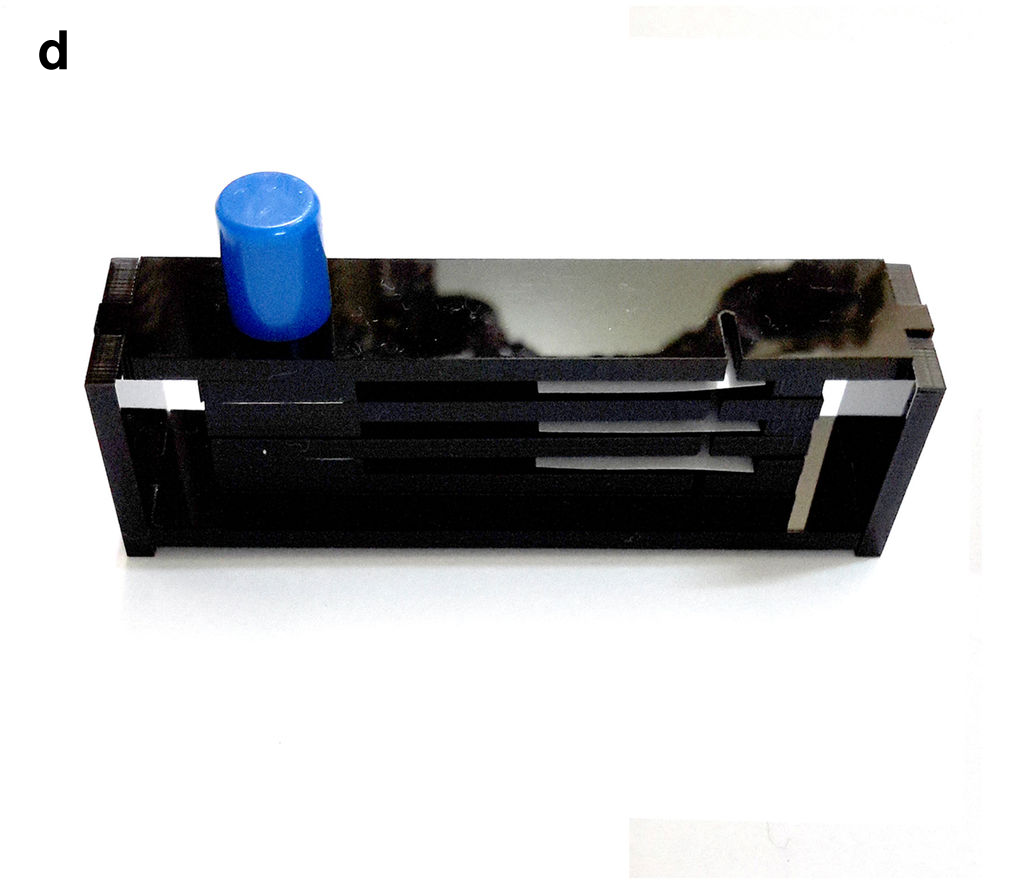
Pinning block (lateral view)

**Figure 3e.**
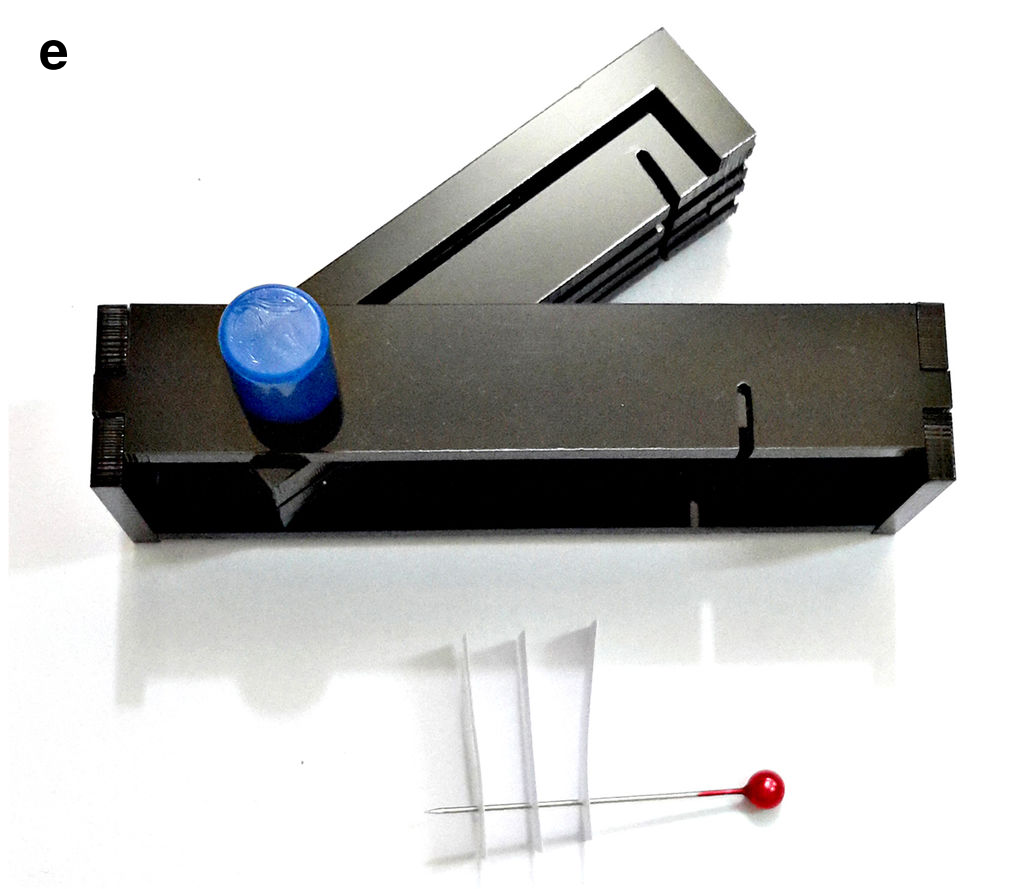
Pinning block (top view)

**Table 1.** Comparison of the important features of available pinning block on the market.

PB* features	**Mechanical PB**	**Traditional wooden stair-step**	**Flexible stair-step**	**Hardwood**	**rectangular wooden**	**rectangular steel**	**extravagant model**
**Applicability**	**NEW**	always	sometimes	often	very often	always	rarely
**Cost**	$5 .00	$3.00	$19	$6.45	$6.35	$39.00	No Price
**Material**	Plexiglas	Wood	Plastic	Wood	Wood	Steel	Silicone
**Precision**	Excellent	Very Good	Fair	Good	Very Good	Excellent	Poor
**Process Labeling**	1-Step	3-Step	3-Step	5-Step	3-Step	5-Step	3-Step
**Quality**	Good	Very Good	Poor	Very Good	Very Good	Excellent	Good
**Duration**	Short	Average	Fair	High	Fair	High	Fair
**Weight**	Light	Average	Light	Average	Average	Heavy	Average
**Final analysis**	**Excellent**	**Very Good**	**Poor**	**Good**	**Very Good**	**Excellent**	**Fair**
