# Supplementary figures and images for: An Upgrade Pinning Block: A Mechanical Practical Aid for Fast Labelling of the Insect Specimens
Source: Biodivers Data J. 2017 Oct 9;(5):e20648. doi: 10.3897/BDJ.5.e20648 (PMC5665011; doi:10.3897/BDJ.5.e20648)

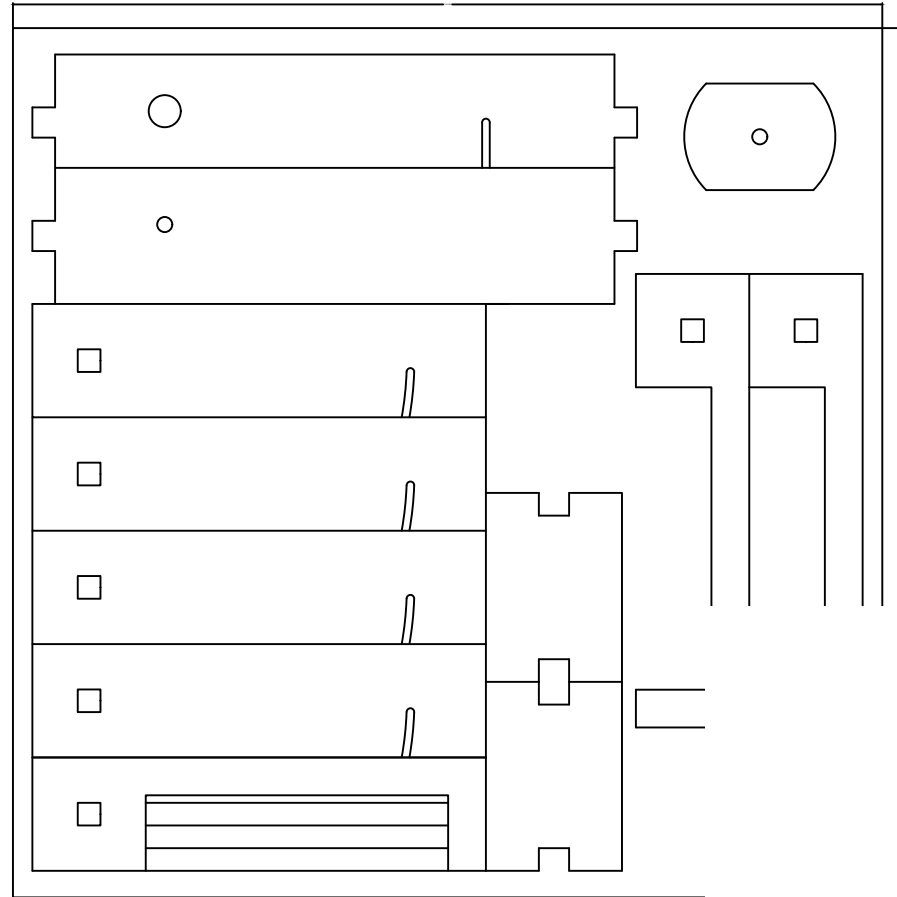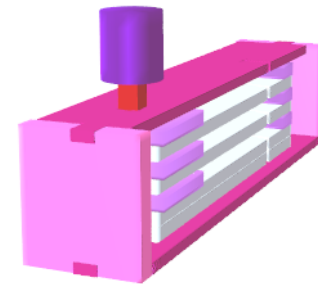

Supplement: Supplementary material 3 — Interactive model [file bdj-05-e20648-s003.pdf]
